# Changes in glucose metabolism among recipients with diabetes 1 year after kidney transplant: a multicenter 1-year prospective study

**DOI:** 10.3389/fendo.2023.1197475

**Published:** 2023-06-21

**Authors:** Jun Bae Bang, Chang-Kwon Oh, Yu Seun Kim, Sung Hoon Kim, Hee Chul Yu, Chan-Duck Kim, Man Ki Ju, Byung Jun So, Sang Ho Lee, Sang Youb Han, Cheol Woong Jung, Joong Kyung Kim, Hyung Joon Ahn, Su Hyung Lee, Ja Young Jeon

**Affiliations:** ^1^ Department of Surgery, Ajou University School of Medicine, Suwon, Republic of Korea; ^2^ Department of Transplantation Surgery and Research Institute for Transplantation, Yonsei University College of Medicine, Seoul, Republic of Korea; ^3^ Department of Surgery, Yonsei University Wonju College of Medicine, Wonju Severance Christian Hospital, Wonju, Republic of Korea; ^4^ Department of Surgery, Jeonbuk National University College of Medicine, Jeonju, Republic of Korea; ^5^ Department of Internal Medicine, School of Medicine, Kyungpook National University, Kyungpook National University Hospital, Daegu, Republic of Korea; ^6^ Department of Surgery, Yonsei University College of Medicine, Seoul, Republic of Korea; ^7^ Department of Surgery, Wonkwang University Hospital, Iksan, Republic of Korea; ^8^ Department of Internal Medicine, College of Medicine, Kyung Hee University, Seoul, Republic of Korea; ^9^ Department of Internal Medicine, Inje University Ilsan Paik Hospital, Goyang, Republic of Korea; ^10^ Department of Surgery, Korea University College of Medicine, Seoul, Republic of Korea; ^11^ Department of Internal Medicine, Bong Seng Memorial Hospital, Busan, Republic of Korea; ^12^ Department of Surgery, Kyung Hee University School of Medicine, Seoul, Republic of Korea; ^13^ Department of Endocrinology and Metabolism, Ajou University School of Medicine, Suwon, Republic of Korea

**Keywords:** kidney transplantation, type 2 diabetes mellitus, glucose tolerance test, immunosuppression therapy, insulin resistance, insulin secretion

## Abstract

**Background:**

Diabetes mellitus is a common and crucial metabolic complication in kidney transplantation. It is necessary to analyze the course of glucose metabolism in patients who already have diabetes after receiving a transplant. In this study, we investigated the changes in glucose metabolism after transplantation, and a detailed analysis was performed on some patients whose glycemic status improved.

**Methods:**

The multicenter prospective cohort study was conducted between 1 April 2016 and 31 September 2018. Adult patients (aged 20 to 65 years) who received kidney allografts from living or deceased donors were included. Seventy-four subjects with pre-transplant diabetes were followed up for 1 year after kidney transplantation. Diabetes remission was defined as the results of the oral glucose tolerance test performed one year after transplantation and the presence or absence of diabetes medications. After 1-year post-transplant, 74 recipients were divided into the persistent diabetes group (n = 58) and the remission group (n = 16). Multivariable logistic regression was performed to identify clinical factors associated with diabetes remission.

**Results:**

Of 74 recipients, 16 (21.6%) showed diabetes remission after 1-year post-transplant. The homeostatic model assessment for insulin resistance numerically increased in both groups throughout the first year after transplantation and significantly increased in the persistent diabetes group. The insulinogenic index (IGI_30_) value significantly increased only in the remission group, and the IGI_30_ value remained low in the persistent diabetes group. In univariate analysis, younger age, newly diagnosed diabetes before transplantation, low baseline hemoglobin A1c, and high baseline IGI_30_ were significantly associated with remission of diabetes. After multivariate analysis, only newly diagnosed diabetes before transplantation and IGI_30_ at baseline were associated with remission of diabetes (34.00 [1.192–969.84], *P* = 0.039, and 17.625 [1.412–220.001], *P* = 0.026, respectively).

**Conclusion:**

In conclusion, some kidney recipients with pre-transplant diabetes have diabetes remission 1 year after transplantation. Our prospective study revealed that preserved insulin secretory function and newly diagnosed diabetes at the time of kidney transplantation were favorable factors for which glucose metabolism did not worsen or improve 1 year after kidney transplantation.

## Introduction

Post-transplant diabetes mellitus (PTDM) is a common and crucial metabolic complication with increasing mortality and cardiovascular disease rates in kidney transplantation (1–3). Previously, new-onset diabetes after transplantation (NODAT) was adopted and implied only diabetes was diagnosed post-transplant. Therefore, NODAT had the shortcoming that there was a chance to exclude undiagnosed diabetes at pre-transplant screening, which is impractical for many transplant centers (4). For comprehensive management of diabetes post-transplant, the term PTDM was re-introduced on behalf of NODAT at an international consensus meeting (5). Because of the difficulty in finding undiagnosed diabetes at pre-transplant screening, it is known that the incidence of PTDM is approximately 10 to 30% (6, 7).

PTDM is caused by many factors before and after surgery, and among them, elements that can be modified and elements that cannot be modified are mixed (8–11). A typical postoperative factor is the use of immunosuppressive drugs such as tacrolimus and steroids, which are risk factors for early PTDM (12). In the case of late PTDM, metabolic causes such as obesity, prediabetes, hyperlipidemia, and insulin resistance are known to have a significant influence (13–15). As such, many studies have conducted research on PTDM. However, most studies have only addressed the risk of developing PTDM in patients without pre-transplant diabetes. Relatively, it is not well known about the natural course of pre-transplant diabetes in recipients who have already undergone a kidney transplant due to diabetic nephropathy or who were diagnosed with diabetes at the time of transplantation. After transplantation, it is thought that hyperglycemia becomes more severe or diabetes is more likely to occur because of the various factors that can cause hyperglycemia. However, on the other hand, there might be a possibility that hyperglycemia or diabetes may not worsen or improve after transplantation. Several studies previously reported how glucose and insulin levels changed in patients after kidney transplantation through the oral glucose tolerance test (OGTT) (16–18). Nevertheless, these previous studies showed meaningful results of OGTT during post-transplant PTDM, but the study populations only consisted of patients without pre-transplant diabetes. Therefore, the authors thought that it was necessary to analyze the changes in glucose metabolism in patients who already have diabetes after receiving a transplant and to understand the characteristics of patients whose glycemic status did not worsen or improve. In this study, we analyzed the results of OGTT follow-up for 1 year for kidney transplant patients who had already been diagnosed with diabetes before transplantation or pre-transplant screening. In addition, we investigated the changes in diabetes status after transplantation and the favorable factors for diabetes remission in recipients with pre-transplant diabetes.

## Materials and methods

### Study population

**Figure 1:**
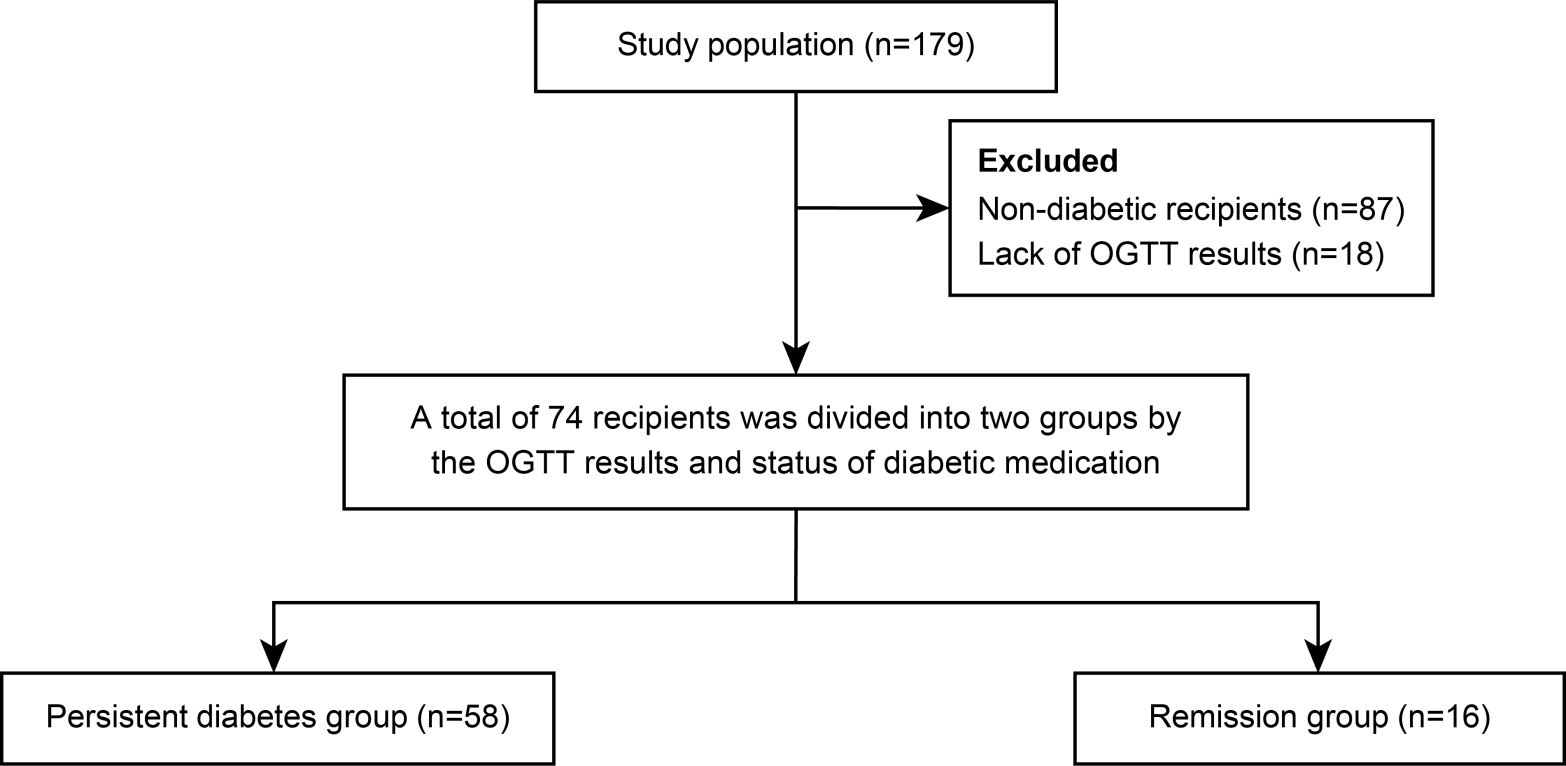
Patient’s flow chart. OGTT, oral glucose tolerance test.

The multicenter prospective cohort study (SERITAM [safety and efficacy of the stepwise reduction of immunosuppression with tacrolimus, mycophenolate mofetil, and basiliximab after kidney transplantation and its effect on glucose metabolism]) was conducted between 1 April 2016 and 31 September 2018. Adult patients (aged 20 to 65 years) who received kidney allografts from living or deceased donors were included (16). The exclusion criteria included multiple organ transplants, a double kidney transplant, or organs donated after cardiac death; previous organ transplantation; ABO incompatibility; crossmatch positivity; and a history of malignancy in the previous 5 years. In total, 179 kidney transplant recipients met the inclusion criteria from 12 centers. All 179 recipients underwent OGTT before transplant and additionally every 3 months until 1 year post-transplant. Among the 179 recipients, 87 were excluded due to being non-diabetic, and 18 were excluded because of insufficient OGTT results during regular visits. After 105 recipients were excluded, the remaining 74 patients with diabetes, including diabetic nephropathy recipients (n = 43) and newly diagnosed diabetes by pre-transplant OGTT (n = 31), were included in the study and 1-year analysis. After 1-year post-transplant, 74 recipients were divided into a persistent diabetes group (n = 58) and a remission group (n = 16) by combining the results of the OGTT performed one year after transplantation and the presence or absence of diabetes medication (**Figure 1**). The patients in the remission group showed negative OGTT results at 1-year post-transplant and met the criterion of not taking antidiabetic drugs or insulin for at least 3 months from 9 months to 1 year after transplantation.

### Immunosuppressive regimen

Previously, the authors described the immunosuppressive regimen in another study, which was conducted by the same protocol (16). The regimen consisted of basiliximab (Simulect^®^, Novartis, Basel, Switzerland) as induction therapy, tacrolimus (TacroBell^®^, Chong Kun Dang, Seoul, Korea), mycophenolate mofetil (MY-REPT®, Chong Kun Dang), and corticosteroids. Basiliximab was administered immediately prior to transplantation and 4 days after transplantation. Tacrolimus was initiated 2 days before kidney transplantation at an initial dose of 0.05 to 0.1 mg/kg. The target tacrolimus trough level was between 5 and 12 ng/ml until 3 months post-transplantation; the target level was then adjusted downward to between 3 and 8 ng/ml until the end of the follow-up period. Corticosteroids were administered intravenously at 500 mg on the day of transplantation and 250 mg on the day after transplantation; they were then gradually tapered to a maintenance dose of more than 5 mg a day of oral prednisone until 6 months post-transplantation. After 6 months, steroids were progressively reduced and stopped in a subset of recipients who were at low immunological risk. In patients who required steroid treatment due to immunological risk, steroids were administered throughout the follow-up period. Mycophenolate mofetil was started within 72 h of transplantation at a dose of 1.0 to 2.0 g/day. In patients who experienced leukopenia or gastrointestinal toxicity, mycophenolate mofetil dose reductions were managed in accordance with a defined protocol; they were guided by the clinical severity and course of the adverse event.

### Assessment of graft function and diabetes

Regular visits were scheduled on the day before transplantation (baseline) and every 3 months until 1 year post-transplant. A full physical examination and routine laboratory results with the tacrolimus level were evaluated at every regular visit. The graft function was assessed using serum creatinine levels and the estimated glomerular filtration rate (eGFR). When acute rejection was clinically suspected, ultrasound‐guided renal biopsies were performed. The diagnosis of acute rejection was classified according to the Banff classification criteria (19).

For assessment of serum glucose and insulin levels, OGTT was performed at baseline and every 3 months after transplantation until the end of the 1-year follow-up period in all patients. The patients were instructed to fast for at least 8 h, and not take oral hypoglycemic agents or inject insulin for 12 h. The patients were then given a drink containing 75 g of glucose mixed in water; plasma glucose and insulin were measured at 0, 30, and 120 min after glucose ingestion. In accordance with American Diabetes Association guidelines, diabetes was diagnosed when one of the following criteria was met: fasting plasma glucose, ≥126 mg/dl; 2-hour plasma glucose, ≥200 mg/dl during OGTT; or requirement of oral hypoglycemic agents or insulin injection (20). Prediabetes was defined as impaired fasting glucose (fasting plasma glucose, 100 to 125 mg/dl) or impaired glucose tolerance (OGTT 2-hour plasma glucose, 140 to 199 mg/dl). Normal glucose, or glucose tolerance, was defined as a fasting plasma glucose of <100 mg/dl and a 2-hour plasma glucose of <140 on the OGTT. Remission of diabetes 1 year after transplantation was defined as normal glucose tolerance or prediabetes on the OGTT without taking anti-diabetic medications.

Additionally, the insulin-based homeostatic model assessment for insulin resistance (HOMA-IR) was used to estimate insulin resistance by means of insulin levels derived from OGTTs, in accordance with an equation reported previously (21). The insulinogenic index (IGI_30_), which was calculated from the insulin and glucose results of OGTTs performed at 0 and 30 min, was used to estimate insulin secretory function (22). HOMA-IR and IGI_30_ were used to check the insulin resistance and secretory function of the patients.

### Statistical analysis

The clinical and OGTT data were collected and assessed at the 3, 6, 9, and 12-month time-points. Categorical variables are expressed as a percentage of derived groups and were assessed using Pearson’s chi-square test and Fisher’s exact test. Continuous variables are expressed as mean ± standard deviation or median with interquartile ranges and were assessed using the Student’s t test and the Mann–Whitney U test. A logistic regression analysis was used to confirm the factors associated with the remission of diabetes. *P*-values <0.05 were considered to indicate statistical significance. All statistical tests were performed using SPSS Statistics version 20.0 (IBM Corp., Armonk, NY, USA).

### Ethics statement

Informed consent was provided to all patients, and the study protocol was approved by the independent Institutional Review Board of each center (approval No. AJIRB-MED-CT4-15-422).

## Results

### Incidence of diabetes remission and basic characteristics of study patients

In total, 74 patients were investigated in this study. The mean age of patients was 52.3 ± 8.7, and 56 (75.7%) patients were male. There were 47 patients with living donor kidney transplants and 37 patients with deceased donor kidney transplants. Diabetes was diagnosed in 43 patients before kidney transplantation, and 31 patients were newly diagnosed with diabetes based on baseline OGTT results. The mean age of donors was 42.9 ± 14.1 years, and 38 (51.4%) donors were male. Twenty-six (35.1%) patients underwent steroid withdrawal therapy 6 months after transplantation. Baseline levels of HbA1c, fasting glucose, and 2-hour glucose, as well as the medication status of all patients, were described in **Supplementary Tables 1, 2**.

**Figure 2:**
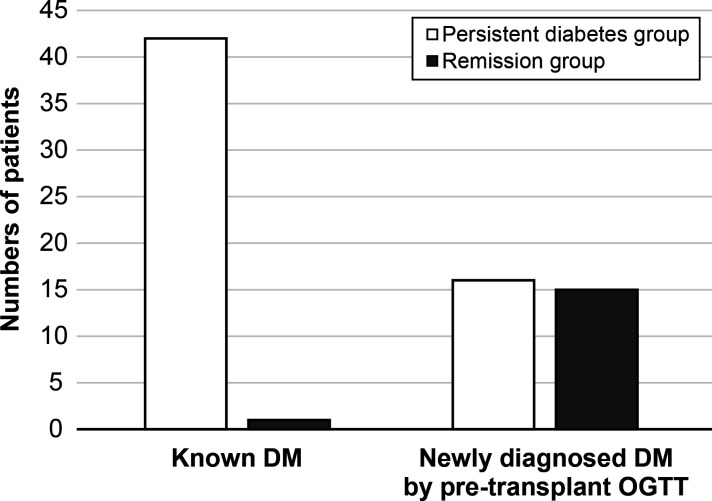
The status of diabetes after 1-year transplantation. DM, diabetes mellitus; OGTT, oral glucose tolerance test.

After 1 year post-transplant, the incidence of diabetic remission was 21.6% (n = 16), and these patients were included in the remission group. The remaining 58 patients were classified as being in the persistent diabetes group. The basic characteristics of the two groups are expressed in **Table 1**. The mean age of patients was significantly older in the persistent diabetes group than in the remission group (54.0 ± 6.8 vs. 46.2 ± 11.8, *P* = 0.021). The baseline hemoglobin A1c (HbA1c) levels were 6.51 ± 1.27% in the persistent diabetes group and 5.28 ± 0.51% in the remission group (*P <*0.001). The other basic characteristics of the two groups were not significantly different. In the persistent diabetes group, there were 16 (27.6%) patients with newly diagnosed diabetes by pre-transplant OGTT. On the contrary, there were 15 (93.8%) patients in the remission group (*P <*0.001). In other words, one year after transplantation, almost half of patients with newly diagnosed diabetes (15/31, 48.4%) achieved remission, and only one patient with pre-existing diabetes (1/43, 2.3%) achieved remission after 1 year of transplantation (**Figure 2**). Additionally, the analysis of the basic and clinical characteristics between newly diagnosed diabetes patients and pre-existing diabetes patients is shown in **Supplementary Table 3.** The characteristics of newly diagnosed diabetes patients were like those of the remission group, as most of the remission group were newly diagnosed diabetes patients (15/16).

**Table 1:** Basic characteristics of the diabetic and remission groups.

	Persistent diabetes group (n = 58)	Remission group (n = 16)	*P*-value
Recipient variables			
Age (yr)	54.0 ± 6.8	46.2 ± 11.8	0.021
Male sex	46 (79.3%)	10 (62.5%)	0.195
Body mass index (kg/m^2^)	24.6 ± 3.8	24.0 ± 4.3	0.598
Body mass index at 1-year post-transplant (kg/m^2^)	23.9 ± 3.1	23.4 ± 3.6	0.517
Dialysis modality			0.093
Hemodialysis	30 (51.7%)	7 (43.8%)	
Peritoneal dialysis	10 (17.2%)	0	
Preemptive transplantation	18 (31.0%)	9 (56.3%)	
Dialysis duration (month)	24.3 ± 31.3	19.1 ± 27.6	0.549
Newly diagnosed diabetes by pre-transplant OGTT	16 (27.6%)	15 (93.8%)	<0.001
Medication for diabetes at baseline	29 (50%)	0	0.002
Insulin	10 (17.2%)		
DPP-4 inhibitor	26 (44.8%)		
Sulfonylurea	12 (20.7%)		
Presence of cardiovascular disease	17 (29.3%)	3 (18.8%)	0.532
HbA1c at pre-transplant (%)	6.51 ± 1.27	5.28 ± 0.51	<0.001
Steroid withdrawal at 6-month post-transplant	21 (36.2%)	5 (31.3%)	0.478
PRA positivity at transplantation			
Class I	5 (8.6%)	1 (6.3%)	0.062
Class II	5 (8.6%)	0	0.579
HLA mismatches	3.1 ± 1.7	2.7 ± 2.1	0.369
Donor variables			
Age (yr)	43.5 ± 14.5	40.7 ± 13.0	0.479
Male sex	30 (51.7%)	8 (50%)	1.000
Type of donation			1.000
Living	37 (63.8%)	10 (62.5%)	
Deceased	21 (36.2%)	6 (37.5%)	

The continuous variable was expressed by mean ± Standard deviation and number of cases with percentages were for the categorical variables.

HbA1c, hemoglobin A1c; HLA, human leukocyte antigen; OGTT, oral glucose tolerance test; PRA, panel reactive antibody; DPP-4, dipeptidyl peptidase-4.

### The results of graft function in the persistent diabetes and remission groups

**Figure 3:**
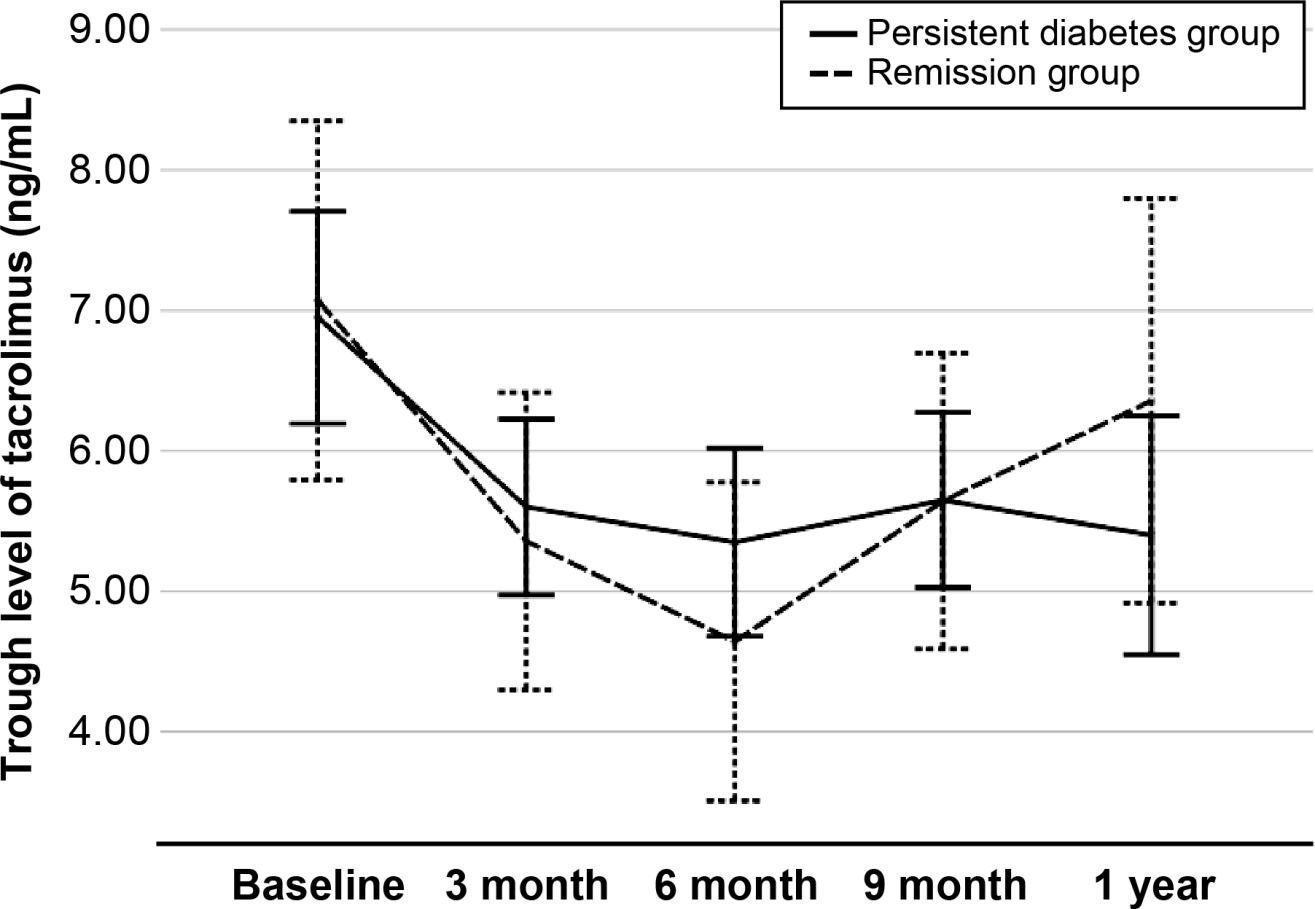
The trough level of tacrolimus during 1-year post-transplantation in two groups.

At 1 year post-transplant, the graft function was compared between two groups. Serum creatinine level was similar in two groups (1.2 ± 0.3 mg/dl vs. 1.3 ± 0.6 mg/dl, respectively, *P* = 0.516), and eGFR was not different in this study (68.5 ± 17.8 vs. 68.7 ± 25.3 ml/min/1.73 m^2^, respectively, *P* = 0.969). The cumulative incidences of biopsy-proven acute rejection was 3 (5.2%) in the persistent diabetes group and 2 (12.5%) in the remission group (*P* = 0.294). There was no graft failure or patient death during the study period (**Table 2**). The levels of tacrolimus at each visit showed no difference between the two groups (**Figure 3**).

**Table 2:** Comparison of post-transplantation clinical parameters and OGTT at 1-year post-transplant between two groups.

	Persistent diabetes group (n = 58)	Remission group (n = 16)	*P*-value
Serum creatinine (mg/dl)	1.2 ± 0.3	1.3 ± 0.6	0.516
eGFR (ml/min/1.73 m^2^)^a^	68.5 ± 17.8	68.7 ± 25.3	0.969
BPAR cumulative incidence at 1-year	3 (5.2%)	2 (12.5%)	0.294
Graft loss or patient death	0	0	1.000
OGTT result at 1-year^b^			<0.001
Normal	1 (1.7%)	5 (31.3%)	
Impaired fasting glucose	1 (1.7%)	3 (18.8%)	
Impaired glucose tolerance	4 (6.9%)	8 (50%)	
Diabetes	52 (89.7%)	0	
Medication for diabetes (yes/no)	51/7	0/16	<0.001
The change of body weight (kg)	-1.3 ± 5.9	-2.0 ± 6.4	0.664

a Chronic Kidney Disease Epidemiology Collaboration method.

b Normal glucose or glucose tolerance was defined as a fasting plasma glucose <100 mg/dl and a 2-hour plasma glucose <140 on the OGTT. Prediabetes was defined as impaired fasting glucose (fasting plasma glucose, 100 to 125 mg/dl) or impaired glucose tolerance (OGTT 2-hour plasma glucose, 140 to 199 mg/dl).

BPAR, biopsy proven acute rejection; eGFR, estimated glomerular filtration rate; OGTT, oral glucose tolerance test.

### The comparison of OGTT results between the persistent diabetes and remission groups

The OGTT was performed at 3-month intervals to determine the changes in diabetes status and glucose metabolism during the post-transplant period. The trajectories of fasting glucose, 2-hour glucose, and HbA1c during 1 year are expressed in **Supplementary Figure 1**. According to the OGTT results and whether subjects were taking antidiabetic medications at 1 year after transplantation, of the 58 recipients in the persistent diabetes group, 52 were in the diabetic range on the OGTT, and six recipients with improved glucose tolerance were taking diabetes medications. In the remission group, there were three (18.8%) IFG, eight (50%) IGT, and five (31.3%) normal results (**Table 2**).

**Figure 4:**
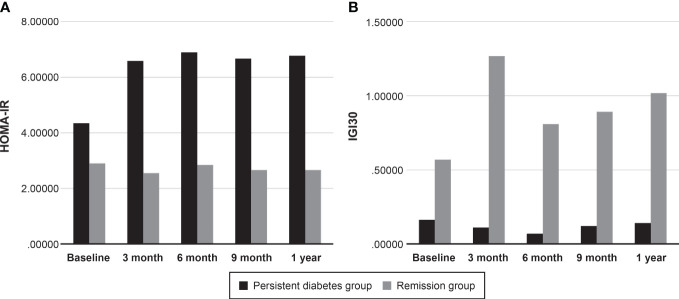
Comparison of insulin secretory function and resistance between two groups. **(A)** HOMA-IR, **(B)** IGI_30_. HOMA-IR, homeostatic model assessment for insulin resistance, IGI_30_, insulinogenic index.

Based on the OGTT results, HOMA-IR and IGI_30_ values were calculated to measure insulin secretory function and resistance (**Table 3**). There was no significant difference in HOMA-IR values between the persistent diabetes group and the remission group at baseline (2.20 [0.89 to 4.62] vs. 1.63 [0.89 to 3.78], *P* = 0.515). Compared with baseline values, HOMA-IR values increased in both groups after transplantation, and in particular, the post-transplant values were significantly higher than baseline in the persistent diabetes group (*P* = 0.007 in the persistent diabetes group and *P* = 0.567 in the remission group) (**Figure 4A**). In addition, the HOMA value of the persistent diabetes group was significantly higher than that of the remission group in the post-transplant tests except for the 9-month test. In the case of IGI_30_ values, the remission group was significantly higher in all tests, including the baseline test, compared with the persistent diabetes group (*P <*0.001 at all times). In the remission group, the IGI_30_ value increased after transplantation, which consistently kept it low in the persistent diabetes group (*P* = 0.101 in the persistent diabetes group and *P* = 0.028 in the remission group) (**Figure 4B**). As a result, the HOMA-IR value increased and the IGI_30_ value remained low after transplantation in the persistent diabetes group. In the remission group, the HOMA value did not significantly increase, and the IGI value after transplantation increased, unlike the persistent diabetes group.

**Table 3:** Comparisons of HOMA-IR and IGI_30_ between two groups.

	Persistent diabetes group (n = 58)	Remission group (n = 16)	*P*-value^a^
HOMA-IR			
Baseline	2.20 (0.89, 4.62)	1.63 (0.89, 3.78)	0.515
3 months	3.49 (1.83, 6.12)	2.26 (1.53, 3.16)	0.032
6 months	4.32 (2.05, 7.61)	2.54 (2.01, 4.12)	0.047
9 months	2.78 (1.59, 5.13)	2.75 (1.38, 3.26)	0.471
12 months	3.30 (1.94, 6.13)	2.02 (1.40, 3.66)	0.041
*P*-value^b^	0.007	0.567	
*Post-hoc* analysis^c^			
Baseline vs. 3 months	0.001	0.669	
Baseline vs. 6 months	0.003	0.495	
Baseline vs. 9 months	0.142	0.495	
Baseline vs. 12 months	0.063	0.597	
IGI_30_			
Baseline	0.09 (0.03, 0.32)	0.50 (0.31, 0.78)	<0.001
3 months	0.06 (0.02, 0.13)	0.93 (0.67, 1.54)	<0.001
6 months	0.08 (0.03, 0.23)	0.87 (0.39, 1.46)	<0.001
9 months	0.07 (0.01, 0.17)	0.85 (0.59, 1.02)	<0.001
12 months	0.08 (0.03, 0.19)	0.81 (0.43, 1.13)	<0.001
*P*-value^b^	0.101	0.028	
*Post-hoc* analysis^c^			
Baseline vs. 3 months	<0.001	0.008	
Baseline vs. 6 months	0.002	0.093	
Baseline vs. 9 months	0.020	0.026	
Baseline vs. 12 months	<0.001	0.018	

a P-values are obtained by using Mann–Whitney U test and corrected using the Bonferroni adjustment.

b P-values are obtained by using the Friedman rank sum test.

c Data are P-values, which are obtained by using Wilcoxon signed-rank test and corrected using the Bonferroni adjustment, which are significant when <0.05.

HOMA-IR, homeostatic model assessment for insulin resistance, IGI_30_, insulinogenic index.

### The factors associated with the remission of diabetes

Logistic regression analysis was performed to evaluate factors associated with remission of diabetes (**Table 4**). Age, newly diagnosed diabetes before transplantation, HbA1c, HOMA-IR, and IGI_30_ at baseline were included in the analysis. In univariate analysis, young age, newly diagnosed diabetes before transplantation, low baseline HbA1c and high baseline IGI_30_ were significantly associated with remission of diabetes. After multivariate analysis, only newly-diagnosed diabetes before transplantation and IGI_30_ at baseline were associated with remission of diabetes (34.00 [1.192–969.84], *P* = 0.039 and 17.625 [1.412–220.001], *P* = 0.026, respectively).

**Table 4:** Logistic regression analysis of factors associated with remission of diabetes.

Variables	Unadjusted OR	*P*-value	Adjusted OR	*P*-value
Age (yr)	0.903 (0.842–0.968)	0.004	0.924 (0.837–1.021)	0.122
Newly diagnosed diabetes	39.37 (4.80–323.0)	0.001	34.00 (1.192–969.84)	0.039
HbA1c (%)	0.167 (0.058–0.482)	0.001	0.378 (0.102–1.406)	0.147
HOMA-IR at baseline	0.930 (0.802–1.078)	0.335		
IGI_30_ at baseline	8.981 (1.897–42.518)	0.006	17.625 (1.412–220.001)	0.026

HbA1c, hemoglobin A1c; HOMA-IR, homeostatic model assessment for insulin resistance; IGI_30_, insulinogenic index.

## Discussion

In this multicenter prospective study, the authors evaluated a case in which recipients with pre-transplant diabetes did not worsen or improve their diabetes status 1 year after kidney transplantation despite taking immunosuppressive drugs that could worsen blood glucose levels after transplantation. As a result of the study, remission of diabetes occurred 1 year after kidney transplantation in 16 out of 74 patients (21.6%). Of the 16 kidney transplant patients whose diabetes had remitted, 15 (93.7%) were those who did not know they had diabetes until they received the transplant but were diagnosed in the pre-transplant baseline OGTT. Patients in the remission group had similar HOMA-IR values and higher IGI_30_ values at baseline and less increased HOMA-IR and more increased IGI_30_ values during the 1-year post-transplant period than those in the persistent diabetes group, suggesting that both insulin resistance and insulin secretory function were better in the remission group patients. In a multivariate analysis to find out the factors that did not worsen or affect the improvement of glucose metabolism, newly diagnosed patients with pre-transplant OGTT and high baseline IGI_30_ values were analyzed as factors that contributed to the remission of diabetes after kidney transplantation.

PTDM is one of the most common metabolic complications in kidney transplant patients (23). The main issue with PTDM is not the risk of developing microvascular disease such as retinopathy, but rather the increased risk of cardiovascular disease and death (24). Furthermore, the previous cohort study reported that 5-year patient and graft survival rates in patients with pre-existing diabetes were significantly low (25). Thereafter, several studies have been conducted on newly developed diabetes after transplantation, identifying and referring to the risk factors for newly developed diabetes in patients without diabetes in advance. There have been several studies on changes in glucose metabolism after OGTT in patients without diabetes before kidney transplantation (17, 26–28). However, as in this study, it is rare to study changes in glucose metabolism and diabetes status by performing OGTT after kidney transplantation in patients with diabetes. Therefore, this study is meaningful to observe the change in diabetes status and changes in serum glucose and insulin in kidney transplant patients who have already been diagnosed with diabetes.

The previous study reported that the incidence of undiagnosed diabetes in kidney transplant candidates without known diabetes was 8.1% (4). In that study, the authors recommended pre-transplant OGTT in non-diabetic patients with high fasting blood glucose before transplantation. In our study, several patients with known diabetes were 61, and the remaining 118 patients were patients without diabetes before pre-transplant OGTT. Of 118 patients, 31 were diagnosed with diabetes by pre-transplant OGTT, and the incidence of undiagnosed diabetes was 26.3% in this study. This result suggested that a relatively large number of kidney transplant candidates could not recognize that they had diabetes. Although the results of this study cannot represent the status of all kidney transplant recipients, it can be inferred that undiagnosed diabetes exists in many kidney transplant candidates, which contributes to the prevalence of PTDM.

In the early period of post-transplantation, immunosuppression was intensively achieved using calcineurin inhibitors (CNIs) and corticosteroids, which can cause diabetes by different mechanisms. In terms of immunosuppression, changes in insulin secretion were primarily associated with CNI, whereas insulin resistance was related to corticosteroid use (29, 30). It is known that disturbance of insulin secretion rather than insulin resistance is the main mechanism for the development of PTDM in the early stages of transplantation (31). In this study, we found that impaired insulin secretion is involved not only in the development of PTDM but also in diabetes remission in patients with pre-existing diabetes. Looking at the IGI_30_ values after transplantation, the IGI_30_ values increased in the remission group compared to the baseline and did not increase in the persistent diabetes group. In particular, the IGI_30_ value of the remission group increased significantly compared to baseline after transplantation. It can be inferred that, in the case of patients with well-preserved insulin secretory function, insulin secretion could be enhanced to compensate for increased insulin resistance when immunosuppressants are administered during the post-transplantation period, thereby preventing the exacerbation of glucose metabolism. It is suggested that preserving insulin secretion is important for both the development and improvement of diabetes.

In terms of OGTT, there was only one chance to perform an OGTT at pre-transplant for diagnosing diabetes in this study. The subjects of this study were patients who were scheduled to undergo kidney transplantation. In the case of deceased donor kidney transplant patients, the preoperative time for OGTT testing was only 1 to 2 days, so only one OGTT could be performed. To overcome some lack of precision, fasting glucose was measured once more before transplant surgery to confirm that they had diabetes among the newly diagnosed diabetes patients, in the case of patients whose only fasting glucose was measured as high in the OGTT result. Nevertheless, there may be limitations to diagnosing diabetes with OGTT. Especially in the remission group, the 2-hour value of seven patients was between 201 and 206 mg/dl. These subjects may have had prediabetes but not diabetes before the transplant. In these patients, it may be correct to mention that glucose metabolism or diabetes status did not deteriorate despite several factors contributing to the rise in blood glucose, such as the use of immunosuppressive drugs, rather than improving 1 year after transplantation.

The glucose metabolism in patients with end-stage renal disease (ESRD) is different from that in patients without ESRD. Dialysis in end-stage renal failure results in changes in glucose metabolism. As insulin resistance improves and insulin excretion increases, each individual’s blood sugar changes due to dynamic contributions from these two factors (32). Kidney transplantation shows similar changes to dialysis in glucose metabolism, and additional effects such as immunosuppressive drugs or kidney gluconeogenesis are added (33). Therefore, kidney transplantation generally has unfavorable effects on glucose metabolism. However, in our study, it was confirmed that the patient’s characteristics—preservation of insulin secretion at baseline and newly diagnosed diabetes—may be more important than the effect of kidney transplantation itself on glucose metabolism. In addition, lifestyle modification in diabetes remission groups like general diabetic patients is also thought to be an important factor for which glucose metabolism did not worsen or improve in kidney transplant patients, and further research is needed.

In this study, the most common cause of ESRD in pre-existing diabetic patients was diabetic nephropathy. Other causes of ESRD in newly diagnosed diabetic patients are hypertensive nephropathy, glomerular disease, and congenital renal disease, which are unknown. Depending on the cause of ESRD, there may be differences in medical care (particularly the use of immunosuppressants) after transplantation. When the cause of ESRD is diabetic kidney disease, a regimen that excludes steroids has been tried in many studies (34–38). Steroids increase the probability of metabolic complications, particularly in those patients. However, in this study, no difference in the use of steroids was observed between patients with diabetes and those without. The steroid withdrawal rate was about 30% in both groups, with no significant difference. Therefore, the effect of steroid use on glucose metabolism after transplantation may not have affected the groups differently in this study.

This study has several limitations. The first is that the number of study subjects is relatively small. It was a multicenter study, and the initial number of subjects was 179. The number of patients had to be small because of the exclusion of patients without diabetes before transplantation and patients with insufficient OGTT results. Although the study population is small, we believe that this number of study subjects can be meaningful because studies that conduct OGTT on kidney transplant patients with diabetes are uncommon. Second, this study included both groups with quite different characteristics according to our study design: a group with diabetic nephropathy and a group with newly diagnosed diabetes. This characteristic was confirmed to be an important factor in glucose metabolism after kidney transplant. That is, most of the patients with remission of diabetes were diagnosed with diabetes through the pre-transplant OGTT, and only one diabetic nephropathy patient showed remission of diabetes 1-year post-transplantation. This is consistent with the fact that remission of diabetes could be closely related to the duration of diabetes. The duration of diabetes is a factor affecting diabetes remission. Unfortunately, the exact duration of diabetes in patients with pre-existing diabetes could not be determined in this study. It was not possible to analyze the association between the duration of diabetes and diabetes remission because there was a lack of data on the duration of diabetes in subjects. However, the high rate of diabetes remission in patients with newly diagnosed diabetes suggests that a shorter diabetes duration may be related to remission. Third, the follow-up period is relatively short. This is because, in the case of a patient who already has diabetes, a period of about 1 year may not be sufficient to observe the pattern of change in blood glucose in kidney transplant patients. Therefore, a long-term follow-up study may be necessary. Lastly, there was a lack of data on recipients, such as their dietary patterns and exercise after transplantation. In addition, the incidence of diabetic retinopathy was not determined in this study. Therefore, we could not evaluate the effect of these factors on glucose metabolism after transplantation. Moreover, the initial body weight of patients may have been incorrect due to fluid retention because they were on dialysis. There could be inaccurate body weights and body mass indices in the study population; that said, most studies that have included dialysis patients probably have this limitation.

In conclusion, our prospective study revealed that in some recipients with pre-transplant diabetes, glucose metabolism did not deteriorate or diabetes status improved 1 year after kidney transplant. They had preserved insulin secretory function or newly diagnosed diabetes at pre-transplant. Considering the mechanism of PTDM that occurs after transplantation, it is reasonable that the ability to secrete insulin to compensate for increased insulin resistance after transplantation is important for glucose metabolism. Newly diagnosed diabetes as a favorable factor for diabetic remission is consistent with the factors in the non-transplant population.

## Data availability statement

The raw data supporting the conclusions of this article will be made available by the authors, without undue reservation.

## Ethics statement

Informed consent was provided to all patients and the study protocol was approved by the independent Institutional Review Board of each center (approval No. AJIRB-MED-CT4-15-422). The patients/participants provided their written informed consent to participate in this study.

## Author contributions

Conceptualization: C-KO and SuL. Data curation: YK, SK, HY, C-DK, MJ, BS, SaL, SH, CJ, JK, and HA. Funding acquisition: C-KO. Investigation: YK, SK, HY, C-DK, MJ, BS, SaL, SH, CJ, JK, and HA. Methodology: C-KO, YK, SK, HY, C-DK, MJ, BS, SaL, SH, CJ, JK, and HA. Writing—original draft: JB. Writing—review & editing: JB, JJ, and SuL. All authors contributed to the article and approved the submitted version.
