# Supplementary material for: Changes in glucose metabolism among recipients with diabetes 1 year after kidney transplant: a multicenter 1-year prospective study
Source: Front Endocrinol (Lausanne). 2023 Jun 21;14:1197475. doi: 10.3389/fendo.2023.1197475 (PMC10325682; doi:10.3389/fendo.2023.1197475)
Supplement: Supplementary file 1 [file DataSheet_1.docx]

Supplementary Material

Changes in glucose metabolism among recipients with diabetes 1 year after kidney transplant: a multicenter 1- year prospective study

Jun Bae Bang, Chang-Kwon Oh, Yu Seun Kim, Sung Hoon Kim, Hee Chul Yu, Chan-Duck Kim, Man Ki Ju, Byung Jun So, Sang Ho Lee, Sang Youb Han, Cheol Woong Jung, Joong Kyung Kim, Hyung Joon Ahn, Su Hyung Lee* and Ja Young Jeon*

*** Correspondence:**

**Ja Young Jeon** twinstwins@hanmail.net

**Su Hyung Lee** [dltngudgs@aumc.ac.kr](mailto:dltngudgs@aumc.ac.kr)

**Supplementary Table 1**. Baseline levels of HbA1c, fasting glucose and 2-hour glucose by OGTT in all patients

| Patients | Group | Newly-diagnosed diabetes | HbA1c (%) | Fasting glucose (mg/dL) | 2-hour glucose (mg/dL) |
| --- | --- | --- | --- | --- | --- |
| 1 | Remission | Yes | 5.4 | 103 | 202 |
| 2 | Remission | Yes | 5.6 | 81 | 241 |
| 3 | Remission | Yes | 4.8 | 158^a^ | 178 |
| 4 | Remission | Yes | 4.7 | 107 | 202 |
| 5 | Remission | Yes | 5.3 | 98 | 202 |
| 6 | Remission | Yes | 6.0 | 92 | 254 |
| 7 | Remission | Yes | 5.2 | 80 | 203 |
| 8 | Remission | Yes | 5.8 | 77 | 269 |
| 9 | Remission | Yes | 5.6 | 107 | 354 |
| 10 | Remission | Yes | 4.7 | 91 | 201 |
| 11 | Remission | Yes | 4.9 | 105 | 247 |
| 12 | Remission | Yes | 4.7 | 83 | 201 |
| 13 | Remission | Yes | 4.8 | 74 | 287 |
| 14 | Remission | Yes | 5.0 | 90 | 206 |
| 15 | Remission | Yes | 5.7 | 89 | 213 |
| 16 | Remission | No | 6.3 | 87 | 180 |
| 17 | Persistent diabetes | Yes | 6.2 | 170 | 392 |
| 18 | Persistent diabetes | No | 5.6 | 117 | 205 |
| 19 | Persistent diabetes | Yes | 6.5 | 94 | 242 |
| 20 | Persistent diabetes | Yes | 4.8 | 112 | 250 |
| 21 | Persistent diabetes | Yes | 6.5 | 90 | 341 |
| 22 | Persistent diabetes | Yes | 6.4 | 91 | 284 |
| 23 | Persistent diabetes | Yes | 4.8 | 71 | 256 |
| 24 | Persistent diabetes | Yes | 4.9 | 74 | 221 |
| 25 | Persistent diabetes | No | 7.1 | 65 | 174 |
| 26 | Persistent diabetes | No | 5.9 | 109 | 178 |
| 27 | Persistent diabetes | No | 6.5 | 113 | 236 |
| 28 | Persistent diabetes | Yes | 5.4 | 98 | 302 |
| 29 | Persistent diabetes | Yes | 6.2 | 94 | 248 |
| 30 | Persistent diabetes | Yes | 5.9 | 146^a^ | 160 |
| 31 | Persistent diabetes | Yes | 4.7 | 111 | 212 |
| 32 | Persistent diabetes | No | 6.1 | 98 | 145 |
| 33 | Persistent diabetes | No | 5.5 | 91 | 242 |
| 34 | Persistent diabetes | No | 5.8 | 111 | 268 |
| 35 | Persistent diabetes | No | 6.5 | 115 | 237 |
| 36 | Persistent diabetes | Yes | 6.5 | 92 | 340 |
| 37 | Persistent diabetes | No | 5.2 | 107 | 170 |
| 38 | Persistent diabetes | Yes | 6.7 | 148^a^ | 149 |
| 39 | Persistent diabetes | No | 9.1 | 185 | 351 |
| 40 | Persistent diabetes | No | 9.1 | 418 | 540 |
| 41 | Persistent diabetes | Yes | 5.3 | 106 | 236 |
| 42 | Persistent diabetes | No | 7.0 | 212 | 324 |
| 43 | Persistent diabetes | Yes | 6.3 | 105 | 309 |
| 44 | Persistent diabetes | No | 4.8 | 125 | 213 |
| 45 | Persistent diabetes | Yes | 7.3 | 76 | 208 |
| 46 | Persistent diabetes | No | 10.4 | 172 | 404 |
| 47 | Persistent diabetes | No | 7.5 | 117 | 403 |
| 48 | Persistent diabetes | No | 7.3 | 218 | 396 |
| 49 | Persistent diabetes | No | 5.9 | 104 | 282 |
| 50 | Persistent diabetes | No | 6.1 | 74 | 236 |
| 51 | Persistent diabetes | No | 7.0 | 72 | 298 |
| 52 | Persistent diabetes | No | 6.3 | 182 | 205 |
| 53 | Persistent diabetes | No | 8.1 | 94 | 272 |
| 54 | Persistent diabetes | No | 5.7 | 125 | 126 |
| 55 | Persistent diabetes | No | 8.8 | 124 | 237 |
| 56 | Persistent diabetes | No | 5.1 | 88 | 272 |
| 57 | Persistent diabetes | No | 6.7 | 222 | 319 |
| 58 | Persistent diabetes | No | 5.9 | 126 | 176 |
| 59 | Persistent diabetes | No | 8.8 | 100 | 206 |
| 60 | Persistent diabetes | No | 6.7 | 128 | 335 |
| 61 | Persistent diabetes | No | 7.0 | 138 | 309 |
| 62 | Persistent diabetes | No | 6.5 | 96 | 189 |
| 63 | Persistent diabetes | No | 7.2 | 144 | 425 |
| 64 | Persistent diabetes | No | 5.2 | 116 | 311 |
| 65 | Persistent diabetes | No | 6.4 | 71 | 297 |
| 66 | Persistent diabetes | No | 6.1 | 46 | 197 |
| 67 | Persistent diabetes | No | 9.7 | 107 | 304 |
| 68 | Persistent diabetes | No | 6.6 | 335 | 551 |
| 69 | Persistent diabetes | No | 6.5 | 151 | 248 |
| 70 | Persistent diabetes | No | 6.5 | 106 | 196 |
| 71 | Persistent diabetes | No | 5.8 | 108 | 326 |
| 72 | Persistent diabetes | No | 6.0 | 102 | 251 |
| 73 | Persistent diabetes | No | 5.6 | 98 | 267 |
| 74 | Persistent diabetes | No | 7.5 | 69 | 274 |

^a^ Fasting glucose was checked twice in patients who diagnosed with diabetes by high fasting glucose

**Supplementary Table 2**. Medications for diabetes during study period in all patients

| Patients | Group | Newly-diagnosed diabetes | Medication at baseline (pre-transplant) | | Medication at 6 month | Medication at 1-year |
| --- | --- | --- | --- | --- | --- | --- |
| 1 | Remission | Yes | 0 | 0 | | 0 |
| 2 | Remission | Yes | 0 | 0 | | 0 |
| 3 | Remission | Yes | 0 | 0 | | 0 |
| 4 | Remission | Yes | 0 | 0 | | 0 |
| 5 | Remission | Yes | 0 | 0 | | 0 |
| 6 | Remission | Yes | 0 | 0 | | 0 |
| 7 | Remission | Yes | 0 | 0 | | 0 |
| 8 | Remission | Yes | 0 | 0 | | 0 |
| 9 | Remission | Yes | 0 | 0 | | 0 |
| 10 | Remission | Yes | 0 | 0 | | 0 |
| 11 | Remission | Yes | 0 | 0 | | 0 |
| 12 | Remission | Yes | 0 | 0 | | 0 |
| 13 | Remission | Yes | 0 | 0 | | 0 |
| 14 | Remission | Yes | 0 | 0 | | 0 |
| 15 | Remission | Yes | 0 | OHA^a^ | | 0 |
| 16 | Remission | No | 0 | OHA | | 0 |
| 17 | Persistent diabetes | Yes | 0 | 0 | | 0 |
| 18 | Persistent diabetes | No | 0 | 0 | | 0 |
| 19 | Persistent diabetes | Yes | 0 | 0 | | 0 |
| 20 | Persistent diabetes | Yes | 0 | 0 | | 0 |
| 21 | Persistent diabetes | Yes | 0 | 0 | | 0 |
| 22 | Persistent diabetes | Yes | 0 | 0 | | OHA |
| 23 | Persistent diabetes | Yes | 0 | 0 | | 0 |
| 24 | Persistent diabetes | Yes | 0 | 0 | | 0 |
| 25 | Persistent diabetes | No | 0 | Insulin | | Insulin |
| 26 | Persistent diabetes | No | 0 | Insulin | | Insulin |
| 27 | Persistent diabetes | No | 0 | Insulin | | Insulin |
| 28 | Persistent diabetes | Yes | 0 | OHA | | OHA |
| 29 | Persistent diabetes | Yes | 0 | OHA | | OHA |
| 30 | Persistent diabetes | Yes | 0 | OHA | | OHA |
| 31 | Persistent diabetes | Yes | 0 | OHA | | OHA |
| 32 | Persistent diabetes | No | 0 | OHA | | OHA |
| 33 | Persistent diabetes | No | 0 | OHA | | OHA |
| 34 | Persistent diabetes | No | 0 | OHA | | OHA |
| 35 | Persistent diabetes | No | 0 | OHA | | OHA |
| 36 | Persistent diabetes | Yes | 0 | OHA | | Insulin |
| 37 | Persistent diabetes | No | 0 | OHA | | OHA |
| 38 | Persistent diabetes | Yes | 0 | OHA | | OHA |
| 39 | Persistent diabetes | No | 0 | Insulin+OHA | | Insulin+OHA |
| 40 | Persistent diabetes | No | 0 | Insulin+OHA | | Insulin+OHA |
| 41 | Persistent diabetes | Yes | 0 | Insulin+OHA | | OHA |
| 42 | Persistent diabetes | No | 0 | Insulin+OHA | | Insulin+OHA |
| 43 | Persistent diabetes | Yes | 0 | Insulin+OHA | | Insulin+OHA |
| 44 | Persistent diabetes | No | 0 | Insulin+OHA | | Insulin+OHA |
| 45 | Persistent diabetes | Yes | 0 | Insulin+OHA | | Insulin+OHA |
| 46 | Persistent diabetes | No | Insulin | Insulin | | Insulin+OHA |
| 47 | Persistent diabetes | No | Insulin | Insulin+OHA | | Insulin+OHA |
| 48 | Persistent diabetes | No | OHA | Insulin | | Insulin |
| 49 | Persistent diabetes | No | OHA | Insulin | | Insulin+OHA |
| 50 | Persistent diabetes | No | OHA | Insulin | | Insulin |
| 51 | Persistent diabetes | No | OHA | Insulin | | Insulin |
| 52 | Persistent diabetes | No | OHA | Insulin | | Insulin |
| 53 | Persistent diabetes | No | OHA | Insulin | | Insulin+OHA |
| 54 | Persistent diabetes | No | OHA | OHA | | OHA |
| 55 | Persistent diabetes | No | OHA | OHA | | OHA |
| 56 | Persistent diabetes | No | OHA | OHA | | Insulin+OHA |
| 57 | Persistent diabetes | No | OHA | Insulin+OHA | | Insulin+OHA |
| 58 | Persistent diabetes | No | OHA | Insulin+OHA | | Insulin+OHA |
| 59 | Persistent diabetes | No | OHA | Insulin+OHA | | Insulin+OHA |
| 60 | Persistent diabetes | No | OHA | Insulin+OHA | | OHA |
| 61 | Persistent diabetes | No | OHA | Insulin+OHA | | Insulin+OHA |
| 62 | Persistent diabetes | No | OHA | Insulin+OHA | | Insulin+OHA |
| 63 | Persistent diabetes | No | OHA | Insulin+OHA | | Insulin+OHA |
| 64 | Persistent diabetes | No | OHA | Insulin+OHA | | Insulin+OHA |
| 65 | Persistent diabetes | No | OHA | Insulin+OHA | | Insulin+OHA |
| 66 | Persistent diabetes | No | OHA | Insulin+OHA | | Insulin+OHA |
| 67 | Persistent diabetes | No | Insulin+OHA | Insulin | | Insulin |
| 68 | Persistent diabetes | No | Insulin+OHA | Insulin+OHA | | OHA |
| 69 | Persistent diabetes | No | Insulin+OHA | Insulin+OHA | | Insulin+OHA |
| 70 | Persistent diabetes | No | Insulin+OHA | Insulin+OHA | | Insulin+OHA |
| 71 | Persistent diabetes | No | Insulin+OHA | Insulin+OHA | | Insulin+OHA |
| 72 | Persistent diabetes | No | Insulin+OHA | Insulin+OHA | | Insulin+OHA |
| 73 | Persistent diabetes | No | Insulin+OHA | Insulin+OHA | | Insulin+OHA |
| 74 | Persistent diabetes | No | Insulin+OHA | Insulin+OHA | | Insulin+OHA |

^a^ OHA included sulfonylurea, DPP-4 inhibitor

OHA=oral hypoglycemic agent

**Supplementary Table 3**. The basic and clinical data between newly-diagnosed and pre-existing diabetes patients

|  | Newly-diagnosed diabetes  (n=31) | Pre-existing diabetes (n = 43) | *P* value |
| --- | --- | --- | --- |
| **Recipients variables** |  |  |  |
| Age (yr) | 49.5 ± 10.4 | 54.4 ± 6.6 | 0.027 |
| Male sex | 21 (67.7%) | 35 (81.4%) | 0.272 |
| Body mass index (kg/m^2^) | 23.5 ± 3.1 | 25.2 ± 4.2 | 0.053 |
| Dialysis duration (month) | 21.7 ± 34.3 | 24.2 ± 27.6 | 0.735 |
| HbA1c at pre-transplant (%) | 5.53 ± 0.72 | 6.74 ± 1.32 | 0.001 |
| Steroid withdrawal at 6-month post-transplant | 7 (22.6%) | 19 (44.2%) | 0.084 |
| HLA mismatches | 2.9 ± 1.8 | 3.1 ± 1.7 | 0.573 |
| **Donor variables** |  |  |  |
| Age (yr) | 40.1 ± 14.3 | 44.9 ± 13.8 | 0.146 |
| Male sex | 15 (48.4%) | 23 (53.5%) | 0.814 |
| Type of donation |  |  |  |
| Living | 21 | 26 | 0.627 |
| Deceased | 10 | 17 |  |
| **Clinical data at 1-year** |  |  |  |
| Serum creatinine (mg/dL) | 1.28 ± 0.56 | 1.18 ± 0.27 | 0.371 |
| eGFR (mL/min/1.73 m^2^)^a^ | 68.5 ± 23.4 | 68.5 ± 16.3 | 0.996 |
| BPAR cumulative incidence | 3 | 2 | 0.644 |
| Graft loss or patient death | 0 | 0 |  |
| The change of body weight (kg) | -1.12 ± 4.96 | -1.8 ± 6.75 | 0.645 |
| **HOMA-IR** |  |  |  |
| Baseline | 3.2 ± 4.5 | 4.5 ± 5.3 | 0.293 |
| 3 months | 3.1 ± 2.1 | 7.7 ± 11.3 | 0.012 |
| 6 months | 3.3 ± 1.8 | 7.8 ± 13.3 | 0.033 |
| 9 months | 3.1 ± 2.6 | 7.5 ± 14.2 | 0.054 |
| 12 months | 3.2 ± 2.3 | 7.8 ± 15.3 | 0.062 |
| **IGI_30_** |  |  |  |
| Baseline | 0.41 ± 0.30 | 0.13 ± 0.52 | 0.013 |
| 3 months | 0.72 ± 0.93 | 0.08 ± 0.14 | 0.001 |
| 6 months | 0.49 ± 0.96 | 0.02 ± 0.69 | 0.021 |
| 9 months | 0.56 ± 0.55 | 0.08 ± 0.54 | 0.001 |
| 12 months | 0.63 ± 0.66 | 0.12 ± 0.34 | 0.001 |

HbA1c = hemoglobin A1c, HLA = human leukocyte antigen, BPAR = biopsy proven acute rejection, eGFR = estimated glomerular filtration rate.

**Supplementary Figure 1**. The trajectories of fasting glucose, 2-hour glucose and HbA1c
